# Brain potential responses involved in decision-making in weightlessness

**DOI:** 10.1038/s41598-022-17234-8

**Published:** 2022-07-29

**Authors:** A. M. Cebolla, M. Petieau, E. Palmero-Soler, G. Cheron

**Affiliations:** 1 Laboratory of Neurophysiology and Movement Biomechanics, Faculté Des Sciences de La Motricité, ULB Neuroscience Institute, Université Libre de Bruxelles, CP640, 1070 Brussels, Belgium; 2 Laboratory of Electrophysiology, Université de Mons, 7000 Mons, Belgium

**Keywords:** Neuroscience, Neurophysiology

## Abstract

The brain is essential to human adaptation to any environment including space. We examined astronauts’ brain function through their electrical EEG brain potential responses related to their decision of executing a docking task in the same virtual scenario in Weightlessness and on Earth before and after the space stay of 6 months duration. Astronauts exhibited a P300 component in which amplitude decreased during, and recovered after, their microgravity stay. This effect is discussed as a post-value-based decision-making closing mechanism; The P300 amplitude decrease in weightlessness is suggested as an emotional stimuli valence reweighting during which orbitofrontal BA10 would play a major role. Additionally, when differentiating the bad and the good docks on Earth and in Weightlessness and keeping in mind that astronauts were instantaneously informed through a visual cue of their good or bad performance, it was observed that the good dockings resulted in earlier voltage redistribution over the scalp (in the 150–250 ms period after the docking) than the bad dockings (in the 250–400 ms) in Weightlessness. These results suggest that in Weightlessness the knowledge of positive or negative valence events is processed differently than on Earth.

## Introduction

The brain is essential to human adaptation to any environment including space. Brain function can be approached by EEG (electroencephalogram) oscillations which underly its dynamics and neural information processing^1–3^. We have previously demonstrated that high-density EEG recordings can be performed on astronauts while being onboard of the International Space Station (ISS) during the execution of different tasks^4,5^. The behavioral adaptation in weightlessness requires a re-weighting of the multiple sensory inputs and motor outputs^6,7^ in such a way that new body-brain neurodynamics^8^ emerge. It has been previously shown that the power of the classical dominant brain alpha rhythm when the eyes are closed is stronger in microgravity than on Earth. This effect was not due to noise, hemodynamic effects, or arousal. It was interpreted as an increasing demand of the central nervous system for avoiding superfluous afferent information in such defiant condition, probably supporting a role in the maintenance of network coherence in the absence of visual information^6^. During a visual-attention task enabling an exploratory watchfulness observational state, it has been shown that astronauts exhibited a stronger power decrease of brain alpha rhythm and that the brain sources accounting for this effect were estimated in the bilateral motor cortex. These observations could be related to the high demands of continuous readjustment or maintenance of an appropriate body posture while free-floating. Additionally, the condition of microgravity was characterized by cerebellar involvement which was interpreted as underlying the correction and error signals necessary for postural stabilization while free-floating, as well as the increased demand to integrate partially reduced or incongruent vestibular information^5^. Moreover, functional brain connectivity analysis on fMRI data recordings performed on Earth after long duration spaceflights of cosmonauts have revealed altered sensorimotor, visual, vestibular and default mode network processing^9–11^.

Weightlessness challenges not only the function and balance of the sensorimotor, arousal, and regulatory systems but also the cognitive and emotional systems^12,13^. Among them, the positive and negative valences, winning or losing, are both the simplest and the strongest events to induce somatic markers^14,15^. These are essential for social processes that subserve all the behavioural health and performance risks^16,17^.

Interestingly, it has been shown that the performance on a visuomotor-tracking task simulating dockings on the ISS, a task very familiar to astronauts^18^ is directly associated to the cognitive performance evaluated by a battery of tests^19^. In the present study, we examined five astronauts’ brain function through their electrical EEG brain potential responses related to their decision-making processes during the execution of a docking task in the same virtual scenario on Earth and in Weightlessness. They were tested on Earth before going to the ISS (Earth_before_), while on the ISS (in Weightlessness) and on Earth after having been to the ISS (Earth_after_). “We hypothesize that the amplitude of the P300 component, known to be sensitive to motivational outcomes (Johnston et al.^28^ ; Keil et al.^29^), will decrease in Weightlessness after the execution of a docking task. According to previous evidence (Kennerley et al.^30^; Rushworth et al.^31^), we also hypothesize that the involvement of the orbitofrontal cortex will be major in Weightlessness for underlying such re-evaluation of the emotional stimuli valence. In this line, we also investigate the P300 modulations and related sources when differentiating between the bad and the good docks on Earth and in Weightlessness, keeping in mind that astronauts were instantaneously informed through a visual cue of their good or bad performance (the terms visual feedback will be used here with this meaning).

## Results

### P300 modulation in weightlessness

**Figure 1:**
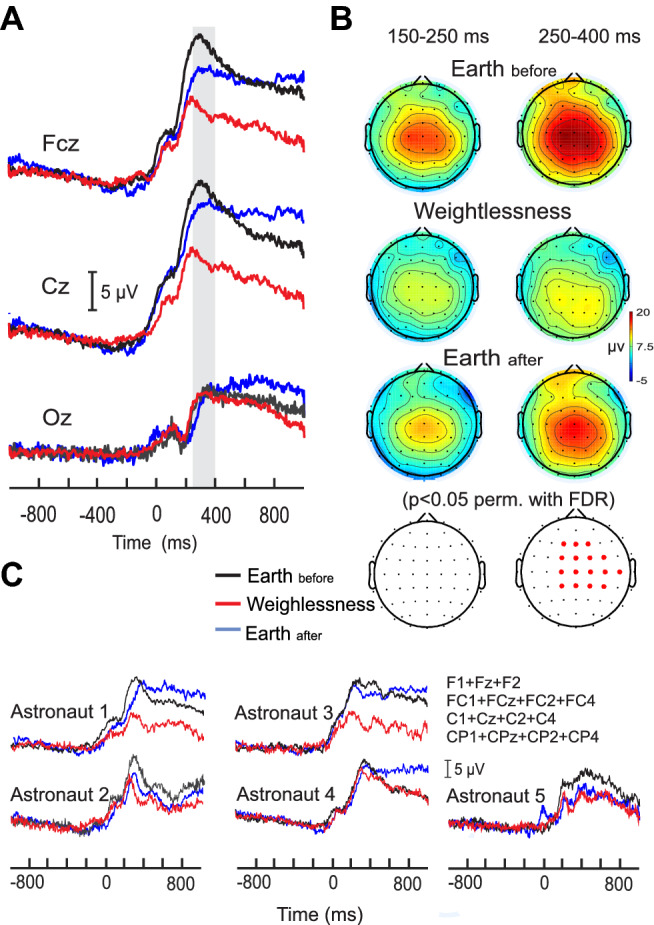
P300 modulation in weightlessness. (**A**) Grand average of the ERP with respect to the press button (for docking) of the five astronauts, on Earth before (black lines), in Weightlessness (red lines) and on Earth after (blue lines), in FCz, Cz and Oz electrodes. The grey bar highlights the 250–400 ms period where significant differences were found for the full array of electrodes as illustrated in (**B**). (**B**) Grand average of the topographical voltage distribution and the statistical differences of the three conditions (Earth_before_, Weightlessness and Earth_after_). (**C**) ERP traces for each of the five astronauts for the three conditions Earth_before_ (black lines), Weightlessness (red lines) and Earth_after_ (blue lines). Each trace is the averaged signal of the significant electrodes obtained by the topographical statistical analysis in (**B**).

In both on-the-ground and in-weightlessness conditions, the event-related electrical potential (ERP) was characterized by a clear positivity centrally localized over the scalp and peaking at around 300 ms after the button press. Figure 1B illustrates the topographical voltage distribution calculated on the full electrodes array across the three conditions for the five astronauts. It is observed that the magnitude of such central positivity strongly diminished in the Weightlessness condition, compared to Earth_before_, and that it regained its magnitude at Earth_after_ (Earth_before_ > in Weightlessness < Earth_after_). The topographical statistical maps showed that the central modulation of electrical potential occurred during the 250–400 ms after the button press and not before this period. In Fig. 1A the grand averaged ERP traces of FCz and Cz electrodes illustrate such potential modulation in the three conditions (FCZ: 19.5 ± 3.3 µV at 319,1 ± 50.6 ms for E_before_ , 11.1 ± 2.4 µv at 306.7 ± 59.5 ms for Weightlessness and 16.1 ± 3.5 µV at 333.6 ± 43.9 ms for E_after._ Cz: 21.2 ± 133.9 µV at 313.3 ± 50.1 ms for E_before,_ 12.7 ± 3.4 µV at 260.8 ± 41.9 for Weightlessness, 19.7 ± 4.9 at 324.2 ± 52.9 ms for E_after_). In contrast, grand averaged ERP of Oz electrode showed similar magnitudes across the three conditions (9.0 ± 6.4 µV at 311.6 ± 30.3 ms for E_b,_ 9.1 ± 2.7 µV at 306.2 ± 44.3 ms for W, 8.8 ± 2.1 µV at 312.9 ± 39.8 ms). Considering the significant electrodes revealed by the statistical maps, individual ERPs for each astronaut are illustrated in Fig. 1C where it is shown that the amplitude decrease of the ERP during Weightlessness with respect to Earth_before_ took place in every astronaut and that it was accompanied by a significantly delayed peak latency in Weightlessness (*p* = 0.05 for Earth_before_ vs Weightlessness; *p* = 0.04 for Weightlessness vs Earth_after_. The related individual amplitude and latency peak values are reported in the Supplementary Table 1). In this line, astronauts 1 and 3 displayed a similar pattern of ERP amplitude modification along with the conditions with ERP voltage diminution in Weightlessness followed by ERP voltage recovery in Earth_after_. Astronaut 4 also showed voltage decrease and voltage recovery in Weightlessness and after space stay respectively but with the particularity that this alteration was very narrowed to the P300 peak. Finally, the ERP traces from Astronauts 2 and 5 suggest a lack of-amplitude recovery following space stay (for related peak values see Supplementary Table 1 and for additional ERP traces of three EEG electrodes for each astronaut see Supplementary Fig. 1). Interestingly and despite individual features, every astronaut presented an ERP voltage decrease from Earth_before_ to Weightlessness conditions in the 250–400 ms period after the button press which produced the docking.

**Figure 2:**
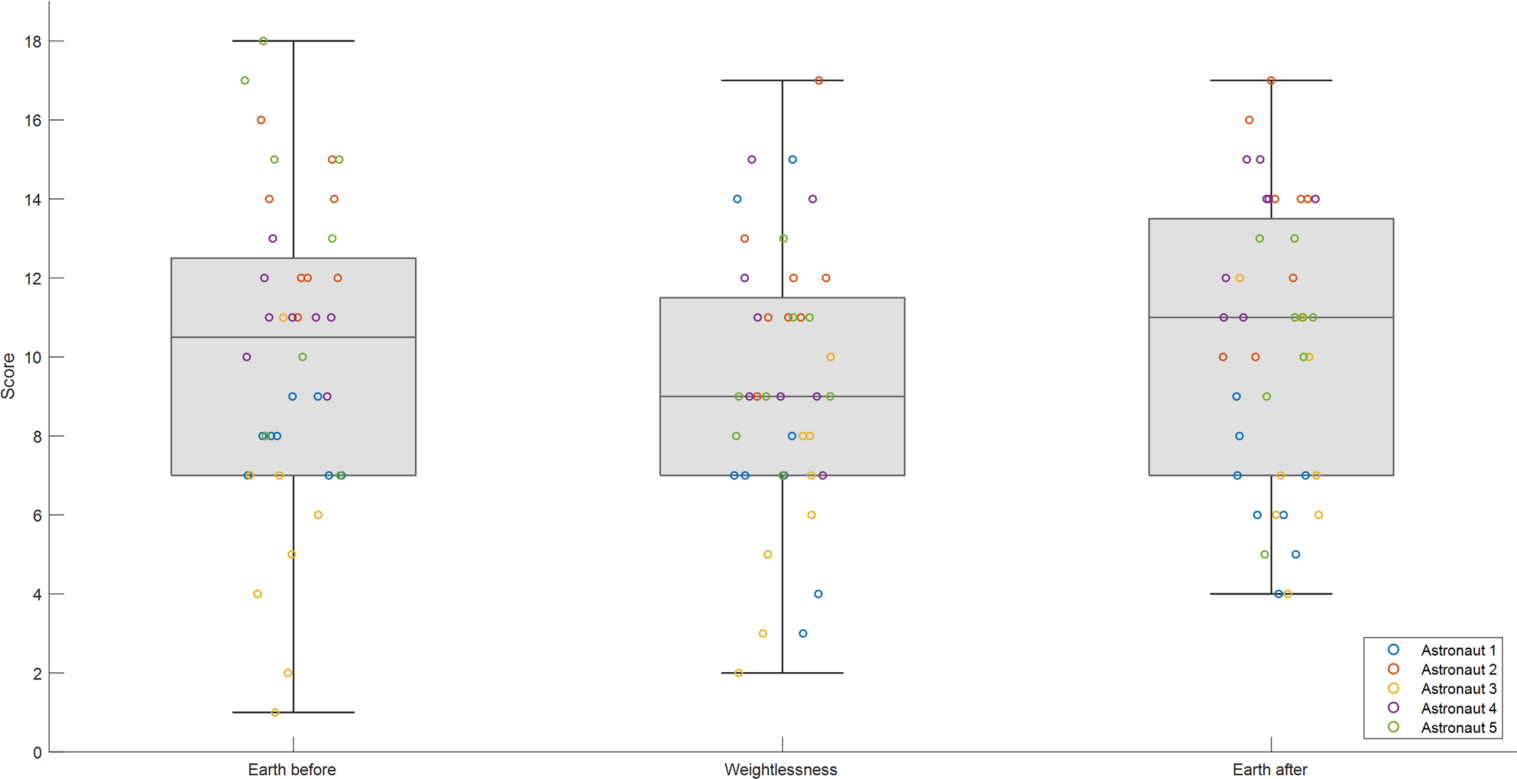
Scores on the docking task. Scores over a value of 20 (because 20 trials) for each of the 4 series per session (two per condition, Earth_before_, Weightlessness and Earth_after_). Boxes represent the inter-quartile range between the 25 and 75 percent quartiles, the horizontal line inside the box indicates the median, the vertical lines mark the minimum and the maximum scores). Individual scores in blue, orange, yellow, violet and green cercles for Astronauts 1, 2, 3, 4 and 5 respectively.

Regarding the astronauts’ performance on the docking task, a tendency (but not significant with nonparametric Friedman Test, *p* = 0.180) of lower scores in Weightlessness was observed (medians of 10.5 for Earth_before_, 9 for Weightlessness, and 11 for Earth_after_ over a maximum score of 20 for each of the 4 series of 20 dockings per session) as illustrated by the boxplots representation in Fig. 2 (boxes represent the inter-quartile range between the 25 and 75 percent quartiles, the horizontal lines inside the box indicate the median, the vertical lines mark the minimum and the maximum scores. For individual score numeric values see Supplementary Table 2.

### Middle frontal gyrus, frontopolar BA10 main involvement in weightlessness’ P300

**Figure 3:**
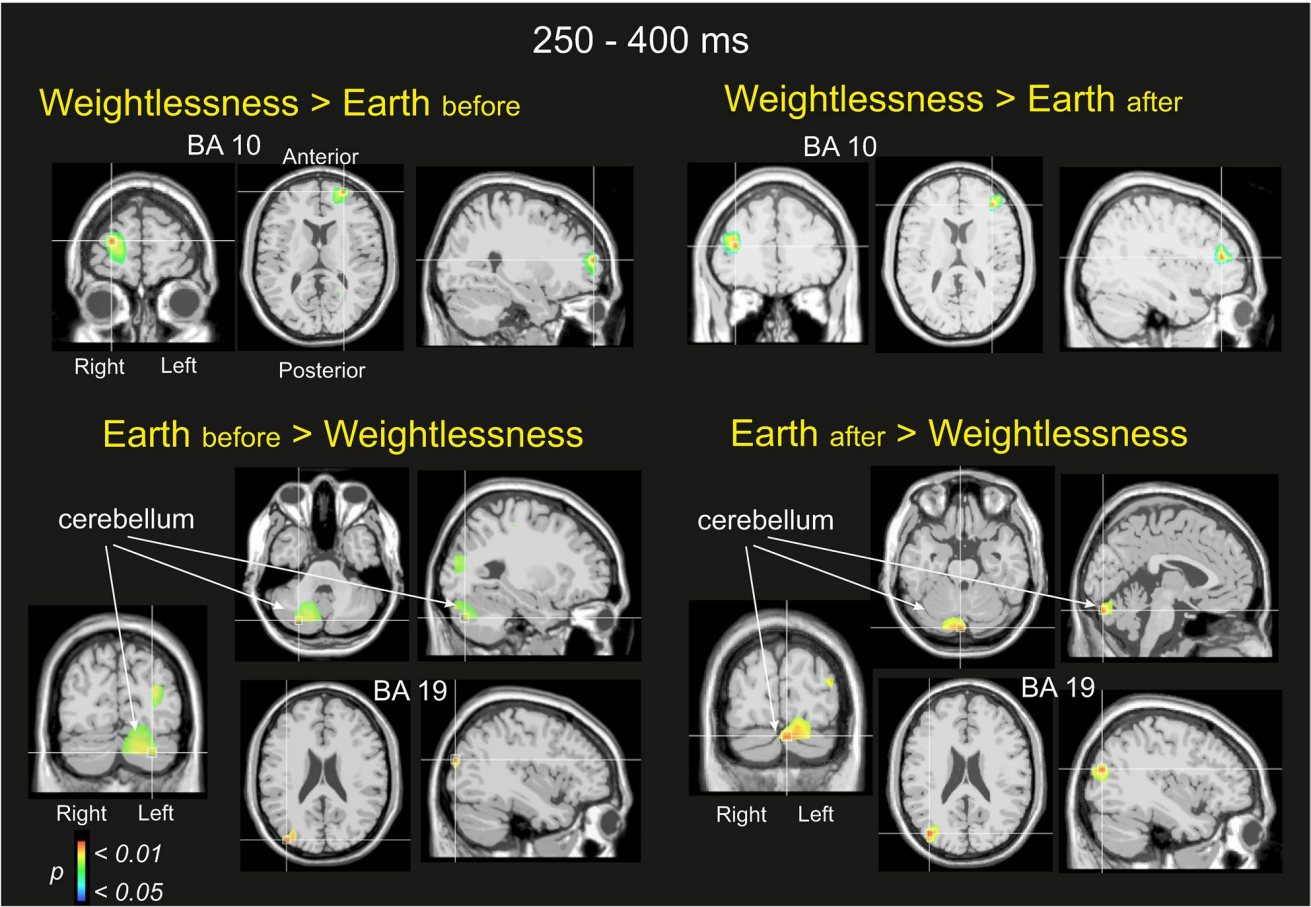
Sources estimations during the P300 component (250–400 ms period). Nonparametric statistical maps calculated on the five astronauts for the Weightlessness > Earthbefore and Weightlessness > Earthafter contrasts, in the upper part of the figure and Earthbefore > Weightlessness and Earthafter > Weightlessness in the lower part of the figure. Note major B10 activation during Weightlessness (for both contrasts > Earthbefore or after), and cerebellum and BA 19 major activations on Earth (indifferently whether Earthbefore > Weightlessness or Earthafter > Weightlessness).

It is well recognized that the potential topographies at the scalp level cannot directly account for their specific brain generators because of pitfalls associated with the mixing of multiple cortical processes by volume conduction^20^. Therefore, to estimate the brain sources that explain the potential modulation that has been previously measured on the topography of the ERPs, we modelled their brain generators by using the swLORETA inverse method^21,22^ in the time domain for all the participants during the period of 250 to 400 ms after the button press. The upper part of Fig. 3 illustrates the nonparametric statistical maps of the sources accounting for the Weightlessness condition contrasted to the Earth_before_ and Earth_after_ conditions separately (Weightlessness > Earth_before_ on the left, and Weightlessness > Earth_after,_ on the right). Interestingly, the model revealed a single and same generator in the right middle frontal gyrus as the main source explaining Weightlessness > Earth_before_ (BA10, maximum at 24, 54, 18) and Weightlessness > Earth_after_ contrast (BA10, maximum at 36, 35, 17).

This indicated that BA10 involvement characterized the topographic voltage modulation occurring in Weightlessness during the 250–400 ms following the button press. The modelling of the sources accounting for both the Earth_before_ > Weightlessness and the Earth_after_ > Weightlessness contrasts (lower part of Fig. 3) revealed two same brain areas for these two contrasts localized in the left cerebellum (posterior lobe, − 26, − 72, − 31 and − 1, − 81, − 24 for Earth_before_ > Weightlessness and the Earth_after_ > Weightlessness contrasts respectively) and the left extrastriate cortex. (BA19, − 36, − 87, 14 and BA19, − 36, − 79, 15 for Earth_before_ > Weightlessness and the Earth_after_ > Weightlessness contrasts respectively). This indicated that cerebellum and BA19 characterized the topographic voltage modulation indistinctly on Earth before or after the space stay, during the 250 to 400 ms following the button press.

### Modulation of brain processing in weightlessness starts sooner after successful than failed performance

**Figure 4:**
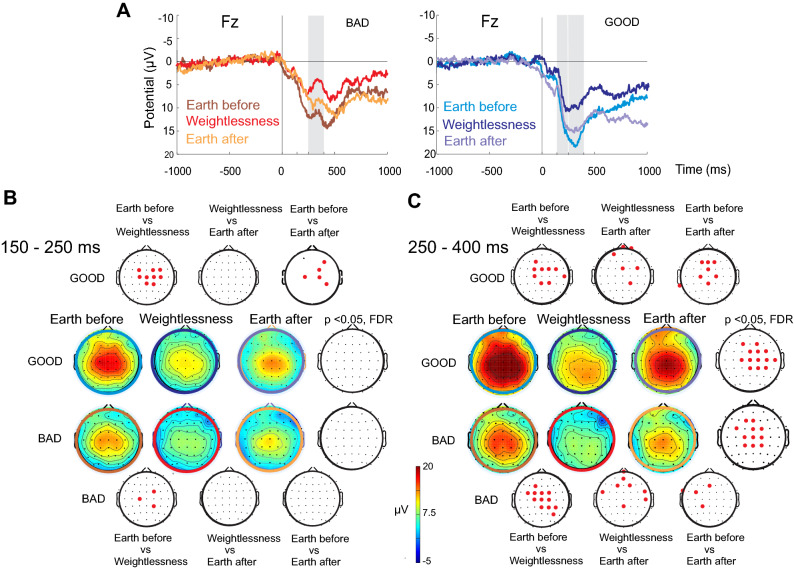
ERPs and topographical voltage distribution for the BAD and GOOD dockings (and related visual feedback: spaceship center yellow or blue colored). (**A**) ERPs for the BAD (on the left) and for the GOOD (on the right) dockings across the three conditions. Bars colored in grey are placed during the periods of interest (250–400 ms on the left; and 150–250 ms and 250–400 ms on the right). (**B**) and (**C**) Topographical voltage distribution of the GOOD and BAD dockings and statistical (permutations, FDR corrected) contrasts for the 150–250 ms (on the left) and 250–400 ms (on the right) periods of interest. Note that the topographical voltage distribution consistently changed in the central area before (in the 150–250 ms period) for the GOOD but not for the BAD dockings when comparing Earthbefore versus Weightlessness. There were no changes between Weightlessness and Earthafter conditions. (**C**) Both GOOD and BAD topographical distributions were modified from Earthbefore to Weightlessness, from Weightlessness to Earthafter and when comparing Earthbefore versus Erathafter in the 250–400 period. In (**B**) and (**C**) colorful circles around the scalp topographies keep the same color code that ERP traces in (**A**).

In our paradigm, the button press (or docking) was instantaneously (at around 3 ms) followed by a colored visual event providing information of either the good (blue) or bad (yellow) performance. In order to investigate whether the good and the bad visual information processing could be differentiated between Weightlessness and Earth conditions (Earth_before_, Earth_after_), the total of EEG epochs centered to the press button event were split into two subgroups. Figure 4A on the left, illustrates the grand averaged ERP at one representative electrode (Fz) of the bad performance and related visual feedback, and Fig. 4 on the right, the grand averaged ERP at the same electrode of the good performance-related visual information, for the three conditions (150–250 ms and 250–400 ms periods are highlighted in grey) (for the individual numeric values of amplitude and latency peaks see Supplementary Table 3). Note a polarity reversal of the P300 component around the 250–400 ms period in the bad performance-related feedback (which corresponds to the feedback-related negativity described in the literature as the feedback stimulus to incorrect responses ^23^; Fig. 4A, on the left). Statistical differences across conditions were calculated in the voltage topographical maps (Fig. 4B and C) which showed that when splitting into good or bad performance visual feedback subgroups, the voltage modulation already started in the 150–250 ms period after the docking but that this was only observed for the visual feedback of the good performance trials from Earth_before_ to Weightlessness with a clear central scalp area localization (Fig. 4B). No consistent topographical potential modulation was observed for the visual feedback of the bad performance in any contrast during the 150–250 ms period after the docking. During the later period of 250–400 ms, consistent topographical voltage modulations were observed for both good and bad performance visual feedbacks (Fig. 4C) in most of the contrasts and this was especially unambiguous for the Earth_before_ to Weightlessness contrast which displays the involvement of central (although not identical) scalp areas.

### Cingulate gyrus, hippocampus, and thalamus generators modelled the good versus the bad response in weightlessness following decision making

**Figure 5:**
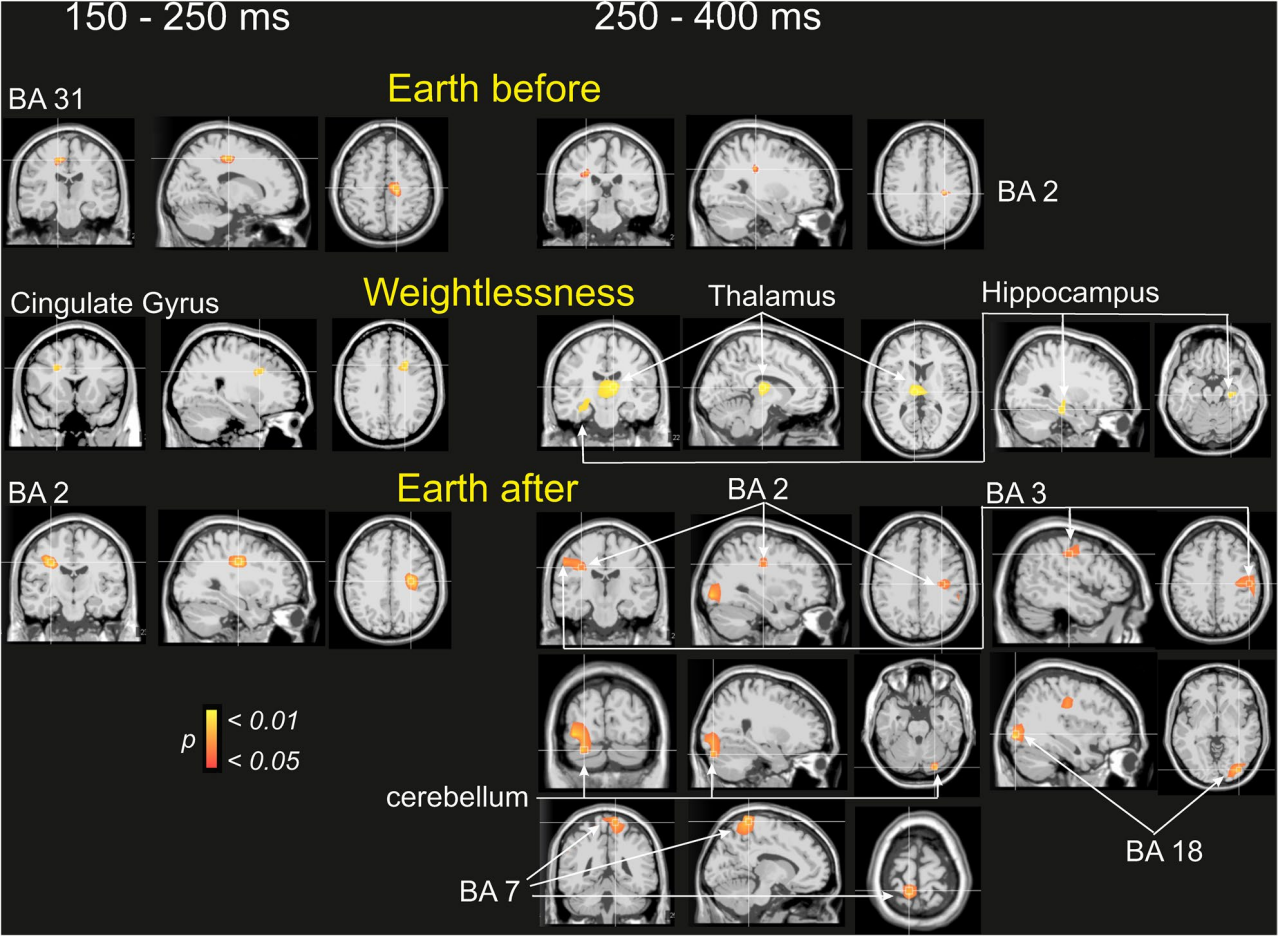
Sources estimations of the GOOD versus BAD (GOOD > BAD) contrasts on the Earthbefore (upper part), Weightlessness (middle part) and Earthafter (lower part) conditions. Nonparametric statistical maps calculated for the five astronauts during the two periods of interest (150–250 ms, on the left; 250–400 ms, on the right).

Avoiding the mixing of multiple cortical processes by volume conduction that might underly the observed different potential scalp topography modulations of the good and the bad performance visual feedbacks, the statistical cartographies at the source level were calculated for the good versus bad performance contrast (good > bad) in the Earth_before_, in Weightlessness and the Earth_after_ conditions and for the two periods of 150–250 ms and 250–400 ms as illustrated in Fig. 5. The model revealed that in the Earth_before_ condition, a generator localized in the right cingulate gyrus (right BA 31, 16, − 23, 44) accounted for the good > bad performance contrast initially during the 150–250 ms period while later it was the right postcentral cortex (right BA2, 30, − 26, 33) during the 250–400 ms period. In the Weightlessness condition, a significant cluster was still localized in the right cingulate gyrus (maximum at 24, 6, 32) during the 150–250 ms while subcortical involvement of the left thalamus (maximum at − 9, − 18, 11) and the right hippocampus (maximum at 30, − 18, − 16) accounted for the good > bad performance contrast during the 250–400 ms period. In the Earth_after_ condition, the model revealed the involvement of the parietal lobe in the postcentral gyrus in the 150 − 250 ms (maximum at right BA2, 32, − 20, 33) and in the 250–400 ms (maxima at right BA2 32, − 20, 33 at right BA3, 53, − 20, 37 and at left BA7, − 10, − 48, 65) periods. In addition, occipital lobe (maximum at right BA18, 37, − 81, − 7) and right cerebellum (maximum at the posterior lobe 26, − 79, − 24) were involved.

## Discussion

Much more than an index of attentional processes, the P300 component ^24,25^ is an endogenous potential independent of sensory modality that was proposed as an essential post-decision closure mechanism ^26^. Under this view and as developed later, the P300 is not responsible for the decision-making process itself but it is responsive to the decision-making process’ outcomes, serving to optimize information processing through the neuromodulatory locus coeruleus–norepinephrine (LC-NE) proposed system ^27^. The astronauts who took part in this study exhibited a P300 component following their decision during the execution of a virtual docking task, and their P300 amplitude decreased during, and recovered after, their microgravity stay. Additionally, the source analysis estimated that the involvement of the frontopolar BA10 characterized the P300 in weightlessness, while in the Earth conditions (before and after the space stay), the involved structures were the left cerebellum and the left extrastriate cortex. Because the visuomotor docking simulation is a very familiar task for astronauts ^19^, it seems reasonable to admit a pre-existent link between the execution of a good docking and rewarding on one hand and, on the other hand, the execution of bad docking and serious risk or cost. It is known that the P300 amplitude is sensitive to motivational outcomes and that a larger P300 amplitude accompanies emotionally valent stimuli, compared to neutral stimuli ^28,29^. Assuming this, the measured P300 here would reflect a post-value-based decision-making closing mechanism, and its amplitude decrease in weightlessness could reflect an emotional stimuli valence reweighting towards neutralization. Orbitofrontal BA10 would be major in such re-evaluation computation ^30,31^.

Trying to better understand the brain response in the re-evaluation of the motivational significance of the good and bad dockings, the EEG trials related to the visual feedback of the good or bad performance were dissociated. We observed that a voltage topographical modulation in Weightlessness started first (in the 150–250 ms period after the docking) for the good but not for the bad visual feedback (related to good and bad performance) and expanded to both the good and the bad ones at a later stage (250–400 ms). The source estimation analysis suggested that such good (versus bad) visual feedback in Weightlessness was initially characterized by the cingulate gyrus and, at a later stage, by the hippocampus and thalamus, while cingulate and postcentral cortex were involved in the Earth_before_, Regarding Earth_after_, postcentral, parietal, occipital cortex and cerebellum were involved. These results suggest that in Weightlessness, the knowledge of positive or negative valence events is processed differently than on Earth.

The present sources estimations are in line with previously well-examined activity and specialized roles of orbitofrontal, cingulate, and parietal cortex, in the neural mechanisms of value computation in value-based decision-making ^31–33^. They also suggest that the hippocampus and thalamus in weightlessness and the cerebellum after the space stay would also play a major role. This can be interpreted under the frame of the LC-NE hypothesis of the P300 proposed by ^34^) and explicated and developed in detail by Nieuwenhuis et al., 2005, proposing that the P300 component reflects a neural system of broadly distributed brain areas sustained and synchronized through the LC-NE system. The latter is known to project throughout the cerebral cortex, thalamus, cerebellum, which is the sole source of NE input to the hippocampus and the neocortex, and to receive afferences from anterior cingulate cortex and orbitofrontal cortex.

Previous findings have reported significant slower and more error visuospatial performance on-orbit than on Earth which was accompanied by event-related brain potentials modifications reflecting diminished attentional resources ^35^. Our present results could not provide a demonstration of significant deterioration of the performance of the virtual docking. However, they suggest that the modification of underlying neural mechanisms can be detected by the EEG brain responses and the P300 component, which is much more than an index of attentional process. Our results suggest that it could reflect a post-value-based decision-making closing mechanism and its amplitude decrease in weightlessness could reflect an emotional stimuli valence reweighting towards neutralization.

The contribution of the cerebellum to the P300 response recorded during the present docking task agrees with the recognized coordinating role exerted by the cerebellum on the frontal, parietal, and hippocampal oscillations during motor and cognitive behaviors ^36^ and with previous ERP studies showing that the amplitude of the P300 was decreased when cathodal tDCS was applied on the cerebellum ^37^. The fact that the cerebellar contribution to the P300 was mainly observed when the docking task was performed on Earth and not when the same task was realized in weightlessness might be explained by the major contribution of the cerebellum to the body control during the free-floating posture adopted by the astronaut during the realization of the docking task (Cebolla et al.^5^). On Earth, the cerebellum integrates the semicircular and otolith signals, providing a neural representation of the head orientation relative to gravity ^38–40^. Because the decision to dock necessitates perceptual stability, the dynamic prediction of the sensory consequences of gravity realized by the internal model of the cerebellum is crucial. It is therefore easily conceivable that the contribution of the cerebellum to decision-making is modified in weightlessness and when the task is performed in a different posture than on earth. Taken together, our results are consistent with the view that the cerebellum builds a dynamic prediction (e.g., internal model) of the sensory consequences of gravity during active self-motion, which in turn enables the preferential encoding of unexpected motion to ensure postural and perceptual stability.

The influence of weightlessness on the dynamic organization of the brain is a central issue for space neuroscience ^4,6,41,42^. Extending EEG in human space exploration can provide evidence of the real brain function adaptations and modifications of astronauts in weightlessness, even before the related behavioral adaptations happen. Having a better understanding of EEG brain responses could allow high-level performers such as astronauts to make adaptations more efficiently. The recent development of human space exploration can benefit from neuroscience research with EEG, both for fundamental and applied purposes.

## Limitations

One limitation of this study is the small sample size of five astronauts. However, high-density EEG recordings in astronauts performed in spaceflight are exceptional and represent a precious tool for directly approaching human brain function in space. Every condition Earth_before_, Weightlessness and Earth_after_, contained the data of two separate sessions that were pooled together for achieving the ERP requirement of having a sufficient and similar number of trials between conditions. As astronauts served as their own controls more repeated measures would have allowed establishing a more robust pre-flight baseline.

It could be also argued that the observed P300 changes in amplitude are due to different EEG recording systems. However, only the postflight sessions of Astronauts 1, 2, and 4 were not recorded with the M.E.E.M.M system and the individual ERP features along the three conditions do not support this criticism.

Astronauts performed the experiment in a seated position on the ground while in free-floating on the ISS. Although no significant differences in the electrophysiological responses between these two positions were previously found in spaceflight (Cheron et al.^6^ and Cheron et al.^4^), we cannot exclude in the present work that different position per se originates specific changes in the amplitude of the P300 amplitude.

Our docking task was not designed as a classical serial choice reaction time task. Astronauts were not asked to perform as fast as possible but as precisely as possible. This limitation prevents direct comparison with the classical P300 of the oddball paradigm and prevents correlation analysis between individual reaction time variabilities and latency modifications of the P300. Interestingly, it has been shown that intraindividual reaction time variability affects P300 amplitude rather than latency and that P300 peak amplitudes become significantly greater for faster relative to slower behavioral responses ^43^.

A general lowering of brain blood oxygenation during microgravity could be signalled as responsible for EEG brain response changes. However, this seems unlikely as such a hemodynamic effect exists during the whole duration of the experiment performed on the ISS and we have previously demonstrated that EEG brain responses modulate after a specific event and not during the baseline periods during the experiment. In this matter, baseline normalization of the signals could be conclusive to rule out the interference of non-specific factors (Cheron et al.^6^, Cheron et al.^4^, Cebolla et al.^5^).

It is important to note that while the structural brain changes are now very well documented ^44,45^, the understanding of the functional brain changes in spaceflight remains in its early stages. The interrelationships between changes in human brain structure and human brain function in spaceflight are hypothetical. It is still conceivable that brain structural changes lead to brain functional changes in microgravity. For instance, it has been suggested that microgravity induces changes in neural membrane viscosity which in turn leads to reduced resting membrane potential, in vitro and in vivo cellular experiments ^46^. Probably, an integrative perspective of the functional mechanisms at the cellular and at the large scale (as EEG), and from imagery techniques will bring a better and unified understanding.

## Conclusion

EEG brain potential responses related to the decision of executing a docking task showed a decreased P300 in astronauts during weightlessness with respect to the Earth conditions. It is discussed that this P300 would reflect a post-value-based decision-making closing mechanism, that its amplitude decrease in weightlessness could reflect an emotional stimuli valence reweighting towards neutralization and that orbitofrontal BA10 would play a major role in such re-evaluation computation.

## Methods

### Participants

This study is a part of a larger project from which previous work has been published in this journal, detailing the main methodological information (Cebolla et al.^5^). Briefly, five male astronauts (54.2 ± 2.6 years old) in excellent health participated in this experiment under the joint European-U.S.A-Canadian Expeditions 20–21 and 34–35, and the joint European-USA Expeditions 26–27 and 30–31, all of them involving 6 months (174.6 ± 19.9 days) on orbit stay. The Ethics Committee of the Faculty of Medicine of the Université Libre de Bruxelles, the European Space Agency Medical Care Committee and the NASA Johnson Space Center Institutional Review Board for Human Testing approved all experimental protocols and procedures, which were performed following the Helsinki Declaration of 1964. All participants gave written, informed consent before starting the experiment. It was required that the participants did not take medications known to affect the central nervous system during the 16 h before the protocol, other than sleep medication the night before if usually taken by the subject. Before each session, all astronauts slept for more than five hours and less than eight hours and the average time asleep was not different across conditions ^47^.

### Stimuli and task and experimental set-up

**Figure 6:**
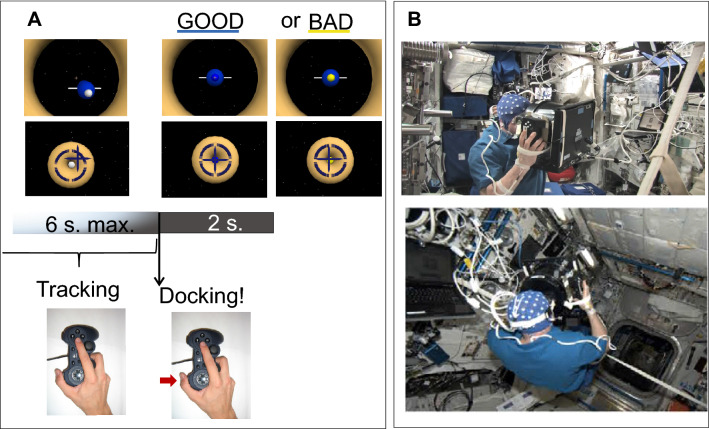
Experimental settings. (**A**) Scheme of the task sequence. After a visuo-motor tracking period for matching positions between spaceship and target (two scenarios), astronauts pressed the button of a joypad to execute the docking. Note the blue centre of the spaceship colored in blue or yellow to inform astronauts of the good or bad performance. (**B**) Astronaut during weightlessness (free-floating) performing the experiment (from Cebolla et al.^6^).

The visuomotor task consisted of matching positions of the virtual spaceship and ISS (docking) employing a joystick (Fig. 6A and B). The visual stimuli were delivered through a virtual scenario displayed on a laptop screen on which a cylindrical tube with a facemask was fitted to remove any external visual cue (Fig. 6B). The screen was centred on the line of sight, at ~ 30 cm from the eyes. The facemask was held firmly in place by a strap that passed behind the head. A joypad was mounted vertically on the right side of the cylindrical tube, allowing the participants to hold on to the entire structure (mask/tube/laptop) with both hands and still manipulate the joystick and press the buttons on the joypad with the right thumb and right index finger. The virtual scenario randomly presented two situations: that of piloting a spaceship towards the ISS; and that of controlling a spaceship from the ISS (Fig. 6A). There were 80 trials (40 for each scenario) administrated in 4 series of 20 trials (or dockings) per session. Each trial lasted 15 s maximum. After a first visuo-attentional period (described in Cebolla et al.^5^) of 6 s duration, the astronauts took control of the spaceship and performed the proper visuomotor task to make the best possible manual adjustment to the spaceship’s trajectory toward the target by pressing on the small joystick with the right index finger. Once the subject considered that he had matched the positions of both crafts as closely as possible and within a limit of 7 s, he pressed a button on the joypad with his thumb for the docking execution. Then instantaneously the center of the spaceship changed from white to blue or yellow, depending on whether the positions were matched acceptably (precision 3 mm) or not. The next trial started 2 s later. The present study focuses on the press button event.

On Earth, the participants were comfortably seated in front of the computer, which rested on a support table. During weightlessness in space flight, they were in a free-floating condition, and they wore a belt around their waist which was attached to straps fixed by metal rings to the right and left racks of the Columbus module of the ISS, to restrain large shifts of trajectory.

After two familiarisation sessions on the ground of one hour and the first session as general rehearsal, each astronaut was tested on the ground before the flight on Earth (“Earth_before_” condition), in weightlessness aboard the International Space Station (ISS) (“Weightlessness” condition) and on the ground after his return on Earth (“Earth_after_” condition). The sessions schedule of this study was at 42.6 ± 0.9 and 28.0 ± 0.4 days before lift-off, at 8.8 ± 1.8 and 54.6 ± 3.7 days of space flight in weightlessness and 3.0 ± 0.4 and 7.0 ± 1.2 days following their landing.

### EEG recordings and pre-processing

The EEG was recorded at a sampling rate of 1116 Hz (0.01–558 Hz band width) using the multi-electrode electroencephalogram mapping module (M.E.E.M.M) from the European physiology module installed in the Columbus module of the ISS, at the European Astronaut Centre (Köln, Germany) or in Star City (Moscow). In addition to the 58 EEG electrodes (from EEG cap with 10–20 electrode system placement), three electrooculograms (for horizontal and vertical EOG), one electrocardiogram, and one electromyogram (from the first interosseous muscle of the right hand) signals were recorded. For some of the post-flight recordings (those of Astronauts 1, 2 and 4) at the Johnson Space Center (Houston), the ANT system (The Netherlands) was used with a sampling frequency of 2048 Hz and with a resolution of 22 bits (71.5 nV per bit). An active-shield cap using 64 Ag/AgCl sintered ring electrodes and shielded co-axial cables (10–20 electrode system placements) was comfortably adjusted to the subject’s head.

All the electrodes were referred to the right earlobe. Scalp electrode impedances were measured and kept below 5 KΩ. Off-line, data treatment was performed using EEGlab ^48^, ASA software (ANT system, The Netherlands) and in-house MATLAB-based tools.

The data were firstly resampled to a 512 Hz and the DC offset was removed. We analysed the single EEG trials (80 trials /session × 5 participants × 2 sessions, per condition) and any artefactual portions of the EEG data were rejected by visual inspection. Synchronous or partially synchronous artefactual activity (mostly blinks) was detected and rejected by independent component analysis (ICA) on continuous data. Epochs were extracted from -1 s before to 1 s after the button press for docking. Visual inspection of the epochs allowed to reject those presenting extreme values and epochs presenting abnormal spectra (0.1–2 Hz ± 50 dB and 20–40 Hz ± 5–100 dB). After the artifact rejection process from 800 epochs per condition, a total of 559 epochs in Earth_before_, 505 epochs in Weightlessness and 620 epochs in Earth_after_ remained from where 308, 247 and 340 epochs corresponded to the good performance of docking for Earth_before_, Weightlessness and Earth_after_ respectively and from where 251, 258 and 280 epochs corresponded to the bad performance of docking for Earth_before_, Weightlessness and Earth_after_ respectively. The significance between the conditions’ ERPs was calculated with non-parametric permutation analysis (*p* < 0.05) and false discovery rate (FDR) was applied for the correction of multiple comparisons (58 electrodes) allowing the representation of statistical topographical scalp maps.

### Source modelling

From the distributed linear solutions available on the commercial ASA software (ANT neuro system), we used swLORETA (standardized weightened Low Resolution Brain Electromagnetic Tomography) ^49,50^ for the brain sources estimation. swLORETA allows accurate reconstruction of surface and deep current sources in simulated data even in the presence of noise and when two dipoles are simultaneously active. The method used here has been described in detail before ^21^. Briefly, we computed the swLORETA solution on the individual ERP topography elicited by the press button during the periods of interest (250–400 ms for assessing the P300 generators; 150–250 ms and 250–400 ms for assessing the good versus bad performance generators). The data were automatically re-referenced to the average reference as part of the LORETA inverse solution analysis and the Boundary Element Model (BEM) was formerly used for solving the forward problem. Voxels (10.00-mm grid size) and the arrangement of the electrodes were placed in registration with the Collins 27 MRI produced by the Montreal Neurological Institute. In ASA software, the corresponding Talairach coordinates are directly accessible for every voxel. The final coordinates (x,y,z, Talairach) reported in the results section correspond to maxima values of the cluster revealed by the statistical analysis. For such source statistical analysis of the contrasts, we used the non-parametric permutation method ^51^ which controls for the false positives that may result from performing multiple hypothesis t-tests (one for each vowel). The probability distribution for testing against the null hypothesis is calculated with the data itself. Paired t-test of swLORETA solutions were used to compare the contrasts. We used the 95th percentile of the calculated permutation distribution for the maximal statistics, which defines the 0.05 level of corrected significance threshold.

## Supplementary Information

Supplementary Information 1.

Supplementary Information 2.

Supplementary Information 3.

Supplementary Information 4.

## Data Availability

All relevant data will be available from the corresponding authors upon request and after approval from the European Space Agency Medical Board (ESA-MB).
