# Supplementary figures and images for: Brain potential responses involved in decision-making in weightlessness
Source: Sci Rep. 2022 Jul 29;12:12992. doi: 10.1038/s41598-022-17234-8 (PMC9338282; doi:10.1038/s41598-022-17234-8)

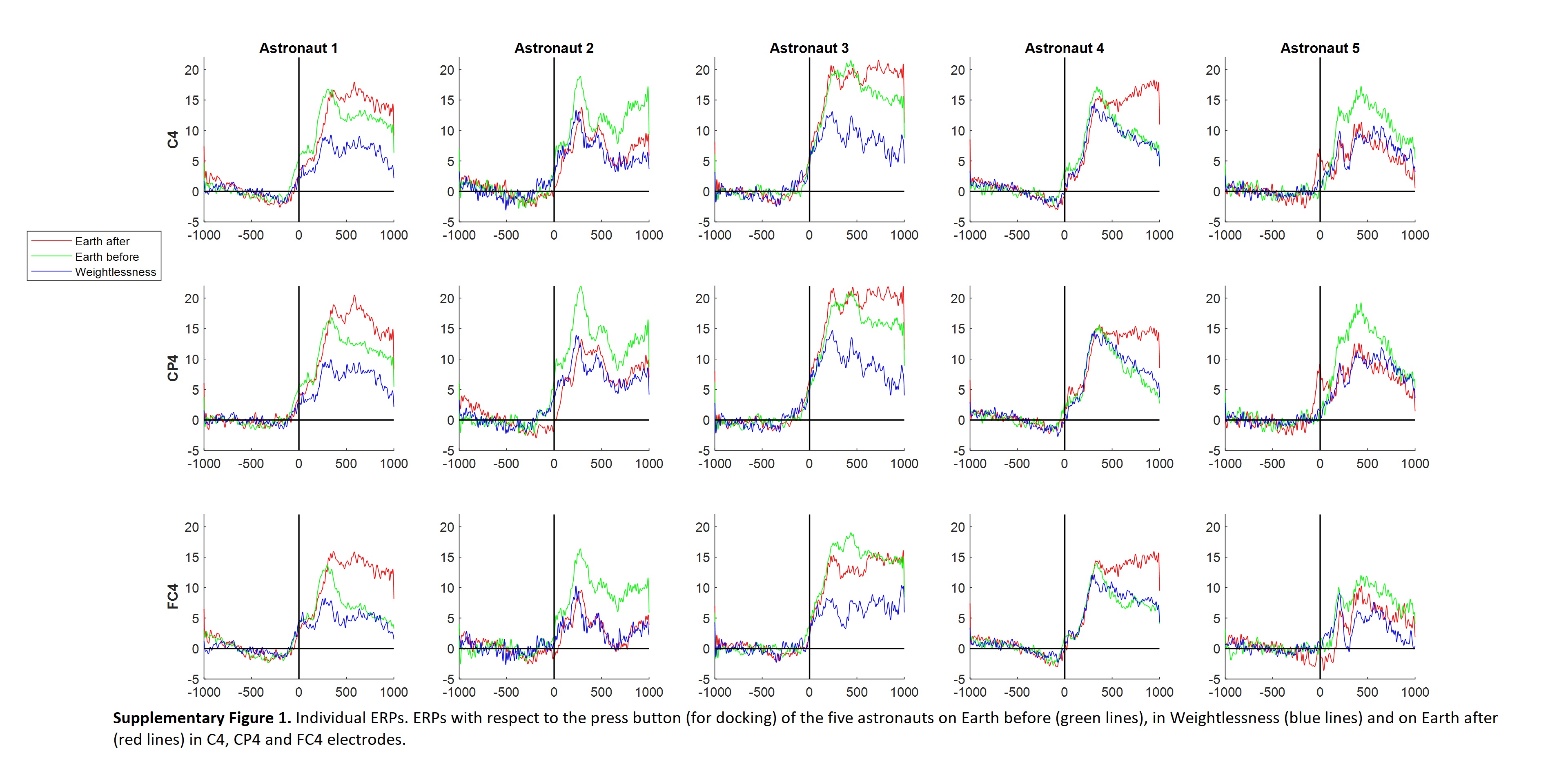

Supplement: Supplementary file 1 — Supplementary Information 1. [file 41598_2022_17234_MOESM1_ESM.jpg]
