# Supplementary material for: Brain potential responses involved in decision-making in weightlessness
Source: Sci Rep. 2022 Jul 29;12:12992. doi: 10.1038/s41598-022-17234-8 (PMC9338282; doi:10.1038/s41598-022-17234-8)
Supplement: Supplementary file 2 — Supplementary Information 2. [file 41598_2022_17234_MOESM2_ESM.pdf]

	Earth before		Weightlessness		Earth after	
	latency (ms)	amplitude (µV)	latency (ms)	amplitude (µV)	latency (ms)	amplitude (µV)
**Astronaut 1**	330,8	19,8	250,0	9,7	363,3	18,8
**Astronaut 2**	261,7	19,9	238,3	13,1	285,2	14,9
**Astronaut 3**	296,9	19,6	244,1	10,4	252,1	18,4
**Astronaut 4**	335,9	17,8	312,5	15,6	365,0	16,5
**Astronaut 5**	415,6	17,1	390,6	10,0	410,7	12,6
**mean**	328,2	18,8	287,1	11,7	335,3	16,2
**sd**	57,2	1,3	65,1	2,5	64,8	2,5

Supplementary Table 1. Individual latency and amplitude peak values of P300 corresponding to ERP traces in Figure 2C.
