# Supplementary material for: Brain potential responses involved in decision-making in weightlessness
Source: Sci Rep. 2022 Jul 29;12:12992. doi: 10.1038/s41598-022-17234-8 (PMC9338282; doi:10.1038/s41598-022-17234-8)
Supplement: Supplementary file 3 — Supplementary Information 3. [file 41598_2022_17234_MOESM3_ESM.pdf]

	Earth before				
	Astronaut 1	Astronaut 2	Astronaut 3	Astronaut 4	Astronaut 5
**Mean**	7,9	13,3	5,4	11,0	12,9
**Median**	8,0	13,0	5,5	11,0	14,0
**Std. Deviation**	0,8	1,8	3,2	1,2	4,1
**Minimum**	7,0	11,0	1,0	9,0	7,0
**Maximum**	9,0	16,0	11,0	13,0	18,0

	Weightlessness				
	Astronaut 1	Astronaut 2	Astronaut 3	Astronaut 4	Astronaut 5
**Mean**	8,1	12,0	6,1	10,8	9,6
**Median**	7,0	11,5	6,5	10,0	9,0
**Std. Deviation**	4,3	2,3	2,7	2,8	1,9
**Minimum**	3,0	9,0	2,0	7,0	7,0
**Maximum**	15,0	17,0	10,0	15,0	13,0

	Earth after				
	Astronaut 1	Astronaut 2	Astronaut 3	Astronaut 4	Astronaut 5
**Mean**	6,5	13,4	7,9	13,3	10,4
**Median**	6,5	14,0	7,0	14,0	11,0
**Std. Deviation**	1,6	2,6	2,8	1,7	2,6
**Minimum**	4,0	10,0	4,0	11,0	5,0
**Maximum**	9,0	17,0	12,0	15,0	13,0

Supplementary Table 2. Numeric score values of the docking task (over a maximum score of 20), in complement to Figure 3 of the manuscript.
