# Supplementary material for: Brain potential responses involved in decision-making in weightlessness
Source: Sci Rep. 2022 Jul 29;12:12992. doi: 10.1038/s41598-022-17234-8 (PMC9338282; doi:10.1038/s41598-022-17234-8)
Supplement: Supplementary file 4 — Supplementary Information 4. [file 41598_2022_17234_MOESM4_ESM.pdf]

BAD	Earth before		Weightlessness		Earth after	
	latency (ms)	amplitude (µV)	latency (ms)	amplitude (µV)	latency (ms)	amplitude (µV)
**Astronaut 1**	298,8	17,8	248,0	9,4	359,4	14,8
**Astronaut 2**	255,9	13,4	232,4	9,7	287,1	9,3
**Astronaut 3**	294,9	15,7	199,2	4,7	287,1	11,1
**Astronaut 4**	398,4	17,2	410,2	14,9	514,6	17,5
**Astronaut 5**	488,3	16,9	210,9	6,8	435,5	13,9
**mean**	347,3	16,2	260,1	9,1	376,7	13,3
**sd**	94,8	1,7	86,0	3,8	98,5	3,2

GOOD	Earth before		Weightlessness		Earth after	
	latency (ms)	amplitude (µV)	latency (ms)	amplitude (µV)	latency (ms)	amplitude (µV)
**Astronaut 1**	289,1	21,1	250,0	11,7	330,1	19,7
**Astronaut 2**	287,1	17,2	228,5	13,4	267,6	13,6
**Astronaut 3**	384,8	22,8	209,0	9,4	226,6	13,9
**Astronaut 4**	334,0	17,5	308,6	18,5	332,0	13,5
**Astronaut 5**	312,5	17,0	205,1	11,7	357,4	15,4
**mean**	321,5	19,1	240,2	12,9	302,7	15,2
**sd**	40,2	2,7	42,2	3,4	53,9	2,6

Supplementary Table 3. Individual latency and amplitude peak values of P300 corresponding to ERP traces of electrode Fz in Figure 5A.
